# Chances and challenges of questionnaires in heart failure care

**DOI:** 10.1007/s12471-024-01922-3

**Published:** 2024-12-10

**Authors:** Aernoud T. L. Fiolet, Mariusz K. Szymanski

**Affiliations:** Department of Cardiology, UMC Utrecht, Utrecht, The Netherlands

There is growing evidence that early identification and treatment of volume overload in patients with heart failure (HF) improve outcomes. In addition, contemporary studies using innovative technologies demonstrate consistent clinical benefit of telemonitoring. However, selecting the appropriate form of telemonitoring remains challenging [1]. Numerous methods exist to evaluate volume status and filling pressures in the outpatient setting. Periodic assessments have proven effective for the early detection of volume overload. Historically, signs of volume overload include rapid weight gain, peripheral oedema and exertional intolerance. However, these parameters show significant variability in predictive value and, particularly, lack sensitivity [2]. Adding clinical assessments, biochemistry, radiology and echocardiography data can enhance diagnostic accuracy.

Automated and invasive methods can improve assessment of filling pressures more precisely in the outpatient clinic. Examples include changes in thoracic impedance or invasive pulmonary artery pressure measurements. While there is no evidence that the former improves clinical outcomes, the latter has demonstrated reductions in HF hospitalisation rates [3, 4]. These methods, however, necessitate a more complex medical infrastructure and are costly, which compromises implementation in routine practice and cost-effectiveness.

Gingele et al. investigated how a patient-reported questionnaire performs compared to the assessment of fluid volume status by a specialised HF nurse [5]. The authors suggest that such a questionnaire could be incorporated into a digital platform, allowing patients to regularly update healthcare providers. In the study, patients were not at home but visiting the hospital with mild to moderate symptoms. The study primarily aimed to establish the test characteristics of the questionnaire rather than to assess its impact on clinical outcomes when implemented.

This is a commendable study design, and the population from this academic hospital appears to align with those in general hospitals. The participants were elderly (mean age 74 years) patients with HF, with reduced and preserved ejection fraction. Most were classified as New York Heart Association functional class I or II. As with any diagnostic study, selecting an appropriate reference standard (‘gold standard’) is crucial. In this case, the nursing specialist’s judgement of fluid status was used as the reference standard, without additional data from biochemistry, radiology, echocardiography or invasive pressure measurements. Sole reliance on physical diagnostic judgement for fluid status is known to be insensitive [6]. However, since this questionnaire is intended for patient self-assessment in the outpatient setting, the absence of additional metrics seems defensible.

The components of the questionnaire were based on prior knowledge without model optimisation. There was considerable variation in the correlation between the questionnaire items and fluid status, suggesting that a shorter questionnaire might yield similar or higher accuracy. Furthermore, the relative weight assigned to each item, which determines the risk score, was chosen arbitrarily based on the researchers’ experience rather than derived from statistical models like linear or logistic regression. The dichotomisation of the risk score offers practical benefits but does not fully reflect the biological and prognostic continuum of volume load and filling pressures in HF patients.

The study’s findings are insightful. The test characteristics of the questionnaire show modest discriminative ability, reflected in a relatively low area under the receiver operating characteristic curve. However, this is comparable to other models used in similar populations. Both the negative and positive predictive values are low, confirming the limited sensitivity of clinical signs such as weight gain. Whether the questionnaire’s current discriminative values are sufficient for self-identification of fluid overload will depend largely on its intended population and its role in the overall monitoring strategy.

The use of questionnaires, in general, poses challenges, including patient compliance (especially with lengthy or repetitive formats) and issues like literacy or inadequate language proficiency in multicultural societies [7]. Nevertheless, this tool offers a straightforward method for identifying patients who may benefit from additional clinical assessments through digital, telephone or in-person consultations. If successful, it could contribute to a cost-effective system for detecting volume overload, facilitating early decongestion and potentially improving patient outcomes. How this questionnaire compares with existing methods for estimating volume status remains to be evaluated. In particular, patient compliance and the proactive involvement required may compete with less labour-intensive options, such as automated data transmission from scales, blood pressure monitors or wearable devices. Future iterations of the questionnaire, its integration into digital platforms and comparisons with other outpatient monitoring methods will be exciting areas for further research.

## References

[1] Scholte NTB, Gürgöze MT, Aydin D, et al. Telemonitoring for heart failure: a meta-analysis. Eur Heart J. 2023;44:2911–26. 37216272 10.1093/eurheartj/ehad280 PMC10424885

[2] Zhang J, Goode KM, Cuddihy PE, Cleland JGF. Predicting hospitalization due to worsening heart failure using daily weight measurement: analysis of the Trans-European network-home-care management system (TEN-HMS) study. Eur J Heart Fail. 2009;11:420–7. 19252210 10.1093/eurjhf/hfp033

[3] Zito A, Princi G, Romiti GF, et al. Device-based remote monitoring strategies for congestion-guided management of patients with heart failure: a systematic review and meta-analysis. Eur J Heart Fail. 2022;24:2333–41. 36054801 10.1002/ejhf.2655 PMC10086988

[4] Brugts JJ, Radhoe SP, Clephas PRD, et al. Remote haemodynamic monitoring of pulmonary artery pressures in patients with chronic heart failure (MONITOR-HF): a randomised clinical trial. Lancet. 2023;401:2113–23. 37220768 10.1016/S0140-6736(23)00923-6

[5] Gingele A, Beckers F, Boyne J, Brunner-La RHP. Fluid status assessment in heart failure patients: pilot validation of the Maastricht decompensation questionnaire. Neth Heart J. 2024;12 10.1007/s12471-024-01921-4. 10.1007/s12471-024-01921-4 PMC11695504 39656355

[6] Kelder JC, Cramer MJ, van Wijngaarden J, et al. The diagnostic value of physical examination and additional testing in primary care patients with suspected heart failure. Circulation. 2011;124:2865–73. 22104551 10.1161/CIRCULATIONAHA.111.019216

[7] Eisele G, Vachon H, Lafit G, et al. The effects of sampling frequency and questionnaire length on perceived burden, compliance, and careless responding in experience sampling data in a student population. Assessment. 2022;29:136–51. 32909448 10.1177/1073191120957102

